# Effects of glycyrrhiza polysaccharides on growth performance, meat quality, serum parameters and growth/meat quality-related gene expression in broilers

**DOI:** 10.3389/fvets.2024.1357491

**Published:** 2024-02-16

**Authors:** Tiyu Li, Weize Qin, Baiyila Wu, Xiao Jin, Rui Zhang, Jingyi Zhang, Liyin Du

**Affiliations:** ^1^College of Animal Science and Technology, Inner Mongolia Minzu University, Tongliao, China; ^2^College of Animal Science, Inner Mongolia Agricultural University, Hohhot, China

**Keywords:** glycyrrhiza polysaccharide, growth performance, meat quality, serum biochemical parameters, gene expression, broilers

## Abstract

With growing restrictions on the use of antibiotics in animal feed, plant extracts are increasingly favored as natural feed additive sources. Glycyrrhiza polysaccharide (GP), known for its multifaceted biological benefits including growth promotion, immune enhancement, and antioxidative properties, has been the focus of recent studies. Yet, the effects and mechanisms of GP on broiler growth and meat quality remain to be fully elucidated. This study aimed to investigate the effects of GP on growth, serum biochemistry, meat quality, and gene expression in broilers. The broilers were divided into five groups, each consisting of five replicates with six birds. These groups were supplemented with 0, 500, 1,000, 1,500, and 2,000 mg/kg of GP in their basal diets, respectively, for a period of 42 days. The results indicated that from day 22 to day 42, and throughout the entire experimental period from day 1 to day 42, the groups receiving 1,000 and 1,500 mg/kg of GP showed a significant reduction in the feed-to-gain ratio (F:G) compared to the control group. On day 42, an increase in serum growth hormone (GH) levels was shown in groups supplemented with 1,000 mg/kg GP or higher, along with a significant linear increase in insulin-like growth factor-1 (IGF-1) concentration. Additionally, significant upregulation of *GH* and *IGF-1* mRNA expression levels was noted in the 1,000 and 1,500 mg/kg GP groups. Furthermore, GP significantly elevated serum concentrations of alkaline phosphatase (AKP) and globulin (GLB) while reducing blood urea nitrogen (BUN) levels. In terms of meat quality, the 1,500 and 2,000 mg/kg GP groups significantly increased fiber density in pectoral muscles and reduced thiobarbituric acid (TBA) content. GP also significantly decreased cooking loss rate in both pectoral and leg muscles and the drip loss rate in leg muscles. It increased levels of linoleic acid and oleic acid, while decreasing concentrations of stearic acid, myristic acid, and docosahexaenoic acid. Finally, the study demonstrated that the 1,500 mg/kg GP group significantly enhanced the expression of *myogenin* (*MyoG*) and *myogenic differentiation* (*MyoD*) mRNA in leg muscles. Overall, the study determined that the optimal dosage of GP in broiler feed is 1,500 mg/kg.

## 1 Introduction

For many years, antibiotics and other medicinal feed additives have played a crucial role in disease prevention and control, as well as in enhancing growth and productivity in livestock and poultry (1). However, the improper use of antibiotics has led to several clinical complications, such as the rise of antibiotic resistance (2), the onset of antibiotic-associated diarrhea (3), the incidence of superinfections (4), and disruptions in the metabolic balance of gut microbiota (5). Consequently, finding green feed additives that can mimic the effects of antibiotics and serve as suitable substitutes has become an essential yet challenging endeavor for researchers and animal breeders. There is substantial evidence suggesting that plant polysaccharides are potential candidates as alternative antibiotic agents (6–8).

Licorice, the dried root and rhizome of legumes like *Glycyrrhiza uralensis* Fisch., *Glycyrrhiza inflata* Bat., and *Glycyrrhiza glabra* L., is known for its multifunctional therapeutic benefits, including Qi movement, relaxing spasms and relieving pain, clearing heat and detoxifying, resolving phlegm and relieving coughs, and reconciling medicinal properties (9). Contemporary research has validated its diverse pharmacological properties, including antioxidative, anti-inflammatory, and antidepressant effects among others (10). Studies have identified the primary active constituents in licorice as saponins, glycyrrhizic acid, flavonoids, and polysaccharides (11). Additionally, extensive research indicates that polysaccharides derived from Chinese herbs not only stimulate animal growth and bolster immune responses but also possess attributes like non-cytotoxicity, minimal side effects, and absence of residues. These characteristics render them promising candidates for developing immunostimulants (12, 13). Glycyrrhiza Polysaccharide (GP), an active polysaccharide derived from licorice, was characterized through chromatographic analysis as containing fucose, rhamnose, arabinose, galactose, glucose, xylose, galacturonic acid and mannuronic acid. The respective contents of these components were determined to be 0.92, 10.12, 6.36, 4.85, 1.34, 0.51, 74.39 and 1.52% (14). Furthermore, GP molecules are abundant in reducing ends, and their conformation predominantly features α-D-pyranose. These structural characteristics enable GP to exhibit various pharmacological activities (15), such as promoting growth (16), enhancing immunity (17), exerting anti-inflammatory (18), and antioxidant effects (19).

Chicken, as one of the predominant meats consumed globally, serves as a major source of animal protein for a significant portion of the human population (20). Consequently, there is a critical need to enhance broiler production efficiency to meet the extensive market demand. Ibrahim et al. demonstrated that *Glycyrrhiza glabra* extract at a concentration of 1 g/kg positively affected broiler growth performance, preserved intestinal integrity, and reduced the shedding of *Campylobacter jejuni* in infected specimens (21). Zhang et al. (22) found that GP dosages ranging from 200 to 1,500 mg/kg consistently lowered the feed-to-gain ratio (F:G) in broilers. However, their study did not elucidate the effects when GP dosage exceeded 1,500 mg/kg, nor did it investigate the mechanisms of growth regulation. Addressing this gap, the present study explores the effect of GP dosages from 500 to 2000 mg/kg on growth performance and probes the expression of growth-related genes in broilers. Furthermore, there is a lack of existing research on the effects of GP on broiler slaughtering performance and meat quality. Hence, this study delves deeper into these aspects, aiming to provide a theoretical reference for the optimal inclusion of GP in broiler diets and enhancing chicken meat quality.

## 2 Materials and methods

### 2.1 Ethical approval

All procedures were approved by the Animal Care and Use Committee of the College of Animal Science and Technology, Inner Mongolia Minzu University (approval code: 2021016). All efforts were made to minimize the suffering of animals.

### 2.2 Animal and experiment design

The current study selected 150 commercial one-day-old, healthy Arbor Acres (AA) broilers. Adhering to the principle of similar body weight (50.07 ± 2.04 g) and balanced gender distribution, the broilers were randomly divided into five treatment groups. The control group was fed a basal diet, while the other groups received the basal diet supplemented with GP at concentrations of 500, 1,000, 1,500, and 2,000 mg/kg. The GP utilized in this experiment was sourced from Weinan Dongjiang Tiancheng Biotechnology Co., Ltd. (Shaanxi, China), and its composition was analyzed by Sanshu Biotechnology Co., Ltd. (Shanghai, China), with the proportions of each component detailed in Table 1. The basal diet met the nutritional requirements as defined by the NRC (23) (Table 2). Each treatment group consisted of five replicates, each containing six broilers. The experiment spanned 42 days, divided into an initial phase (days 1–21) and a later phase (days 22–42).

**Table 1:** Composition analysis and monosaccharide breakdown of GP extract %.

Item	Percentage
Ingredient	
Total sugars	81.73
Moisture	4.41
Ash	2.12
Fiber	1.81
Glycyrrhizic acid	6.15
Flavonoids	3.78
Monosaccharide components	
Arabinose	6.91
Ribose	8.97
Xylose	2.32
Glucuronic acid	3.25
Galactose	11.92
Glucose	28.18
Mannose	14.10
Galacturonic acid	24.35

**Table 2:** Composition and nutrient levels of basal diets (as-fed basis) %.

Item	Feeding phase	
	Day 1 to 21	Day 22 to 42
Ingredient		
Corn	54.00	58.00
Peeled soybean meal	29.00	26.00
Flour	4.00	4.00
Corn protein powder	3.00	2.00
Soybean oil	1.90	1.80
Limestone	1.35	1.40
DCP^1^	1.75	1.80
Hydrolyzed feather meal	1.00	1.00
Common salt	0.25	0.50
Sodium humate	0.20	0.20
SB^1^	0.15	0.10
L-tyrosine	0.10	0.00
Choline chloride	0.10	0.10
DL-methionine	0.02	0.00
L-lysine	0.18	0.10
Premix^2^	1.00	1.00
Calculated nutrient		
ME (Kcal/kg)^1^	2,999	3,009
Dig-Lysine	1.01	0.90
Dig-Methionine	0.31	0.29
Dig-TSAA^1^	0.61	0.57
Dig-Threonine	0.77	0.64
Dig-Tryptophan	0.21	0.19
Ca	1.00	1.00
Total P	0.63	0.62
Non-phytate P	0.43	0.40
Analyzed nutrient		
Crude protein	22.13	20.06

^1^DCP, dicalcium phosphate; SB, sodium bicarbonate; ME, metabolizable energy; TSAA, total sulfur amino acid. ^2^Provided per kg of diet: Vitamin A, 15,000 IU; Vitamin E, 47 IU; Vitamin K, 6 mg; Thiamine, 3 mg; Riboflavin, 9 mg; Pyridoxine, 6 mg; Cobalamin, 0.03 mg; Niacin, 60 mg; D-Pantothenic acid, 16 mg; Folic acid, 1.5 mg; Biotin, 0.06 mg; Choline, 900 mg; Zinc sulfate, 40 mg; Ferrous sulfate heptahydrate, 80 mg; Sodium selenite, 0.15 mg; Calcium iodate, 0.35 mg; Copper (II) sulfate pentahydrate, 8 mg; Manganese (II) sulfate monohydrate, 80 mg.

Single-layer cage housing was employed for the trial. During the first week, continuous 24-h lighting was provided, which was adjusted to 23 h of light with 1 h of darkness starting from the second week. The temperature was maintained at 32–35°C during the brooding phase and was reduced by 1°C every 2 days starting from the first week, stabilizing at 24–26°C by the third week. Humidity levels were controlled between 60 and 70%. Feed and water were provided *ad libitum* throughout the study. At 7 days of age, the broilers were immunized against Newcastle disease with nasal and ocular drops; at 14 days, they were immunized against bursa of Fabricius disease using a similar method; and at 21 days, they were re-immunized against Newcastle disease. The health status of the broilers was regularly monitored, ensuring they remained free from diseases or health complications for the duration of the study.

### 2.3 Sample collection

The experiment spanned a period of 42 days. On days 21 and 42 of the experiment, at 8:00 AM, broilers were weighed on an empty stomach following a 12-h fast, with each replicate serving as a unit. From each group, 5 mL of blood was randomly collected from one broiler per replicate via the wing veins into vacutainer tubes. The blood samples were then centrifuged at 3,500 g for 15 min at 4°C. Serum was separated and stored at −20°C for subsequent analysis of serum indicators. Following blood collection, the same broilers were euthanized through cervical dislocation and exsanguinated. The left pectoralis major muscle and the left bundle-shaped anterior tibial muscle were then excised from the broilers. During sampling, care was taken to avoid compression and to maintain the integrity of the fibers. Samples approximately 1 × 1 × 0.5 cm in size were placed in 5 mL tubes and fixed with 4% paraformaldehyde for later analysis of muscle fiber characteristics. Concurrently, around 2 g of the sample was placed in a 5 mL cryotube, flash-frozen in liquid nitrogen, and stored at −80°C for determining related gene mRNA expression levels. Additionally, 40 g of muscle tissue was wrapped in tin foil and stored at −20°C for assessing fatty acid composition and storage stability. The right pectoral and leg muscles were also fully stripped, weighed, and analyzed for muscle pH, meat color, drip loss, cooking loss, and shear force values.

### 2.4 Measurement of growth performance

The feed offered and feed refused were weighed and recorded daily to calculate the Average Daily Feed Intake (ADFI). Broilers were weighed prior to morning feeding on days 1, 21, and 42 of the trial. The Average Daily Gain (ADG) and Feed-to-Gain Ratios (F:G) were calculated for each group.

### 2.5 Analysis of serum indices

The thawed serum samples were analyzed using an automatic biochemistry analyzer (HITACHI 912; Hitachi, Tokyo, Japan) to determine the concentrations of Total Protein (TP), Globulin (GLB), Alanine Aminotransferase (ALT), Aspartate Aminotransferase (AST), Alkaline Phosphatase (AKP), and Blood Urea Nitrogen (BUN). The levels of Growth Hormone (GH) and Insulin-like Growth Factor 1 (IGF-1) were quantified using chicken-specific ELISA kits (Enzyme-linked Biotechnology, Shanghai, China) in accordance with the manufacturer’s instructions.

### 2.6 Determination of body size and carcass traits

On day 42 of the experiment, measurements of body slant length, tibial length, tibial circumference, hip bone width, keel bone length, chest angle, chest depth, and chest width were conducted in replicates. After exsanguination during slaughter, the weights of the carcass, whole cleaned thorax, half cleaned thorax, pectoral muscle, thigh muscle, abdominal fat, and external gizzard fat were recorded. The fully eviscerated weight, semi-eviscerated weight, breast muscle ratio, thigh muscle ratio, abdominal fat ratio, and lean meat ratio were then calculated. The measurement and calculation methods adhered to the agricultural industry standards of China (24).

### 2.7 Evaluation of meat quality parameters

On day 42 of the experiment, following exsanguination during slaughtering, samples from the breast and thigh muscles were collected for assessing various meat quality parameters.

#### 2.7.1 pH value

The pH meter (Handylab 2, SCHOTT, Mainz, Germany) was calibrated with pH 4.6 and 7.0 buffer solutions. The pH values of the breast and thigh muscles were then measured at 45 min and 24 h post-slaughter, respectively, at a temperature of 4°C.

#### 2.7.2 Meat color

Forty-five minutes post-slaughter, the chroma meter (CR-200, MINOLTA, Tokyo, Japan) was calibrated using a white porcelain tile as per the instruction manual. The meat color of the breast and thigh muscles was measured using the Lab* color system of the chroma meter.

#### 2.7.3 Cooking loss

Approximately 100 g of thigh and breast muscles (W1) were weighed and steamed for 25 min. The muscle samples were then quickly removed from the steamer, allowed to cool at room temperature for 15 min, and reweighed (W2). The cooking loss rate was calculated using the formula:

$$\mathrm{Cooking}\;\mathrm{loss}\;\mathrm{rate}\;\left(\%\right)=\left(W1-W2\right)/W1\times 100\%$$

#### 2.7.4 Drip loss

Meat strips measuring 3 × 2 × 1 cm^3^ were trimmed along the muscle fiber direction and weighed (W3). The strips were then hung vertically inside a sealed plastic container at 4°C, parallel to the muscle fibers. After 24 h, the samples were removed and reweighed (W4). The drip loss rate was calculated as follows:

$$\mathrm{Drip}\;\mathrm{loss}\;\mathrm{rate}\;\left(\%\right)=\left(W3-W4\right)/W3\times 100\%$$

#### 2.7.5 Shear force

Post the determination of cooking loss, three meat strips were cut along the muscle fiber direction from the cooked meat. A dynamometer (C-LM, Northeast Agricultural University, Harbin, China) was used to measure the shear force thrice on each strip. The reported shear force value is the average of these measurements.

#### 2.7.6 Muscle fiber characteristics

After removal from paraformaldehyde, the pectoral and leg muscles were washed twice with 1x PBS solution and trimmed into tissue blocks approximately 1 × 0.5 × 0.3 cm in size. The samples were sequentially dehydrated in increasing concentrations of ethanol (50, 70, 80, 90, 95, and 100%) for 45 min at each concentration. This was followed by immersion in a mixture of ethanol and xylene, and then in xylene solution alone, each for 15 min. Subsequently, the samples were soaked in paraffin at 50–60°C for 4 h, with the wax being changed every 30 min. Embedding was performed using a tissue embedding system (Histostar, Thermo Fisher Scientific, China). Sections of 5 μm thickness were cut perpendicular to the muscle fibers using a paraffin microtome (RM2235, Leica Microsystems, Shanghai, China) and mounted on anti-adhesive slides. Each sample was sectioned six times, with every third section mounted on a single slide. The sections were air-dried at room temperature for 3 h before undergoing Hematoxylin–Eosin (HE) staining using a kit (Solarbio Science & Technology Co., Ltd., Beijing, China), as per the manufacturer’s specifications. The paraffin sections were examined under a compound microscope (RM2235, Leica Microsystems, Shanghai, China). For each replicate, five fields were randomly selected at 10 × 40 magnification, and images were captured for quantitative analysis using ImageJ software. Twenty muscle fibers were randomly selected from each image to measure the average diameter and to count the number of muscle fibers within a grid reticule, representing the average muscle fiber density of the sample.

#### 2.7.7 Storage stability

Pectoral and leg muscle samples were retrieved from storage at −20°C, and the content of thiobarbituric acid reactive substances (TBARS), carbonyl, and sulfhydryl groups was determined using colorimetric methods after 90 days of storage. The absorbance for these measurements was recorded at wavelengths of 532 nm, 370 nm, and 412 nm, respectively. The sampling procedure and testing methods were conducted in strict accordance with the protocols described by Yu (25).

#### 2.7.8 Fatty acid composition

The quantification of fatty acids in chicken meat was conducted using an 8,890-7000D Gas Chromatography–Mass Spectrometry (GC–MS) system (Agilent, United States). This analysis primarily involved the methyl esterification of fatty acids, a critical step in preparing the sample for GC–MS analysis, as delineated in the methodology established by Moyo et al. (26). Post-esterification, the fatty acid methyl esters (FAMEs) were separated on a chromatographic column and identified by comparison to standard chromatograms.

For the GC analysis, the operational parameters were meticulously set. The separation was executed on a DB-Fast Fame 30 m × 250 μm × 0.25 μm chromatographic column, operating at a flow rate of 1 mL/min and a transfer line temperature of 280°C. The temperature programming commenced at 80°C, sustained for 0.5 min, followed by a first gradient increase at a rate of 40°C/min up to 165°C, and held for 1 min. This was succeeded by a second gradient increase of 4°C/min until 230°C, which was maintained for 4 min. The total run time for this analysis was approximately 23 min, followed by a post-run phase at 260°C for 5 min. Additionally, the MS settings included an electron energy of 70 eV, with a solvent delay time of 2 min, and a final run time of 1 min.

### 2.8 Quantitative reverse transcription PCR (RT-qPCR) analysis

The gene expressions of *GH*, *GHR*, *IGF-1*, *IGF-1R*, *IGFBP-1*, *MyoG* and *MyoD* were evaluated using quantitative reverse transcription PCR (RT-qPCR), with β-actin serving as the normalization reference. The details of the primers, based on chicken sequences from GeneBank and synthesized by TaKaRa Biotechnology Co., Ltd., Dalian, China, are provided in Table 3. Leg muscle tissue RNA was isolated using Trizol reagent (Solarbio, Beijing, China). RNA purity and concentration were assessed by NanoDrop spectroscopy (NanoDrop Technologies, Wilmington, DE, United States), and all samples were standardized to 500 ng/μL. RNA integrity was verified through 1.5% agarose gel electrophoresis. cDNA synthesis was carried out following the manufacturer’s protocol (Takara Biotechnology, Dalian, China). RT-qPCR was conducted on a CFX Connect System (Bio-Rad) using a SYBR Green PCR kit (Takara Biotechnology, Dalian, China). The RT-qPCR reaction mixture had a total volume of 20 μL, which included 2 μL of nucleic acid template, 10 μL of 2 × TB Green Premix Ex Taq II (Tli RNaseH Plus), 0.4 μL of ROX Reference Dye (50 ×), 0.8 μL each of forward and reverse primers (10 μM), and 6 μL of nuclease-free water. The thermal cycling conditions for the RT-qPCR amplification were set as an initial denaturation at 95°C for 10 min, followed by 40 cycles of 15 s at 95°C for denaturation and 1 min at 60°C for annealing/extension. The correlation coefficients of all standard curves were > 0.99, and the amplification efficiency ranged from 90 to 110%, thereby validating the use of the 2^–▵▵CT^ method for the calculation of relative gene expression levels (27).

**Table 3:** Primer sequences for RT-qPCR.

Target^1^	Sequence of nucleotide^2^ (5′ to 3′)	Fragment size (bp)	GenBank accession
*GH*	For: TTCAAGAAGGATCTGCACAAGGT	85	NM_204359
	Rev: CTCAGATGGTGCAGTTGCTCTCT		
*GHR*	For: GCGTGTTCAGGAGCAAAGCT	121	NM_001001293
	Rev: TGGGACAGGCATTTCCATACTT		
*IGF-1*	For: GATGCTCTTCAGTTCGTATG	146	NM_001004384
	Rev: TACATCTCCAGCCTCCTC		
*IGF-1R*	For: TTCAGGAACCAAAGGGCGA	158	NM_205032
	Rev: TGTAATCTGGAGGGCGATACC		
*IGFBP-1*	For: GGCAAAGGCTCAGCAGAGAAGTG	119	NM_001001294
	Rev: CAGCGGAATCTCCATCCAGTGAAG		
*MyoG*	For: GCGGAGGCTGAAGAAGGTGA	120	NM_204184
	Rev: CGCTCGATGTACTGGATGGC		
*MyoD*	For: GGAGAGGATTTCCACAGACAACTC	113	NM_204214
	Rev: CTCCACTGTCACTCAGGTTTCCT		
*β-actin*	For: GAGAAATTGTGCGTGACATCA	152	NM_205518
	Rev: CCTGAACCTCTCATTGCCA		

^1^*GH*, growth hormone; *GHR*, growth hormone receptor; *IGF-1*, insulin-like growth factor-1; *IGF-1R*, insulin-like growth factor 1 receptor; *IGFBP-1*, insulin like growth factor binding protein 1; *MyoG*, myogenin; *MyoD*, myogenic differentiation; *β-actin*, beta actin. ^2^For, forward primer; Rev., reverse primer.

### 2.9 Statistical analysis

Data were compiled in Microsoft Excel 2021 and subjected to one-way ANOVA using the GLM procedure in SAS (Version 9.4, SAS Institute Inc., Cary, NC, United States). In experiments assessing growth, slaughtering, meat quality and blood biochemical indicators, linear and quadratic contrasts were used to assess the effects of increasing dietary GP concentrations through orthogonal polynomials. Group comparisons were conducted using Duncan’s multiple comparison test. Data variability was represented as the pooled standard error of means (SEM), and *p* < 0.05 was considered statistically significant.

## 3 Results

### 3.1 Growth performance

The effects of different doses of GP supplementation in the diet on the growth performance of broilers are shown in Table 4. As shown in Table 4, an increase in the GP dosage did not elicit any significant variations (*p* > 0.05) in both ADG and ADFI across all evaluated periods. However, during the intervals of 22 to 42 days and from 1 to 42 days, dietary incorporation of GP at concentrations of 1,000 and 1,500 mg/kg significantly reduced (*p* < 0.05) the F:G ratio. Furthermore, a marked linear trend (*p* < 0.05) and a quadratic response (*p* < 0.05) in the F:G ratio were shown with increasing levels of GP.

**Table 4:** Effect of dietary GP on the growth performance in broilers.

Items	Levels of dietary GP (mg/kg)					SEM	*p*-value		
	0	500	1,000	1,500	2,000		Treatment	Linear	Quadratic
BW (g)									
1 d	49.96	50.83	49.21	50.00	50.33	0.87	0.756	0.973	0.916
21 d	452.12	447.29	448.58	469.54	445.72	13.45	0.837	0.862	0.967
42 d	1913.8	1904.5	2021.6	1997.3	1945.7	35.1	0.149	0.233	0.155
ADG (g/d)									
1–21 d	19.15	18.88	19.02	19.98	18.83	0.64	0.833	0.861	0.961
22–42 d	69.60	69.39	74.90	72.75	71.43	1.67	0.188	0.250	0.188
1–42 d	44.38	44.14	46.96	46.36	45.13	0.83	0.144	0.234	0.155
ADFI (g/d)									
1–21 d	30.67	29.12	29.56	30.51	29.72	0.69	0.504	0.821	0.739
22–42 d	137.11	133.79	137.48	131.19	134.48	2.30	0.350	0.316	0.581
1–42 d	83.89	81.45	83.52	80.85	82.10	1.36	0.484	0.343	0.572
F:G									
1–21 d	1.60	1.54	1.55	1.53	1.58	0.04	0.627	0.589	0.336
22–42 d	1.97^a^	1.93^ab^	1.84^bc^	1.80^c^	1.88^abc^	0.03	0.027	0.030	0.009
1–42 d	1.89^a^	1.85^ab^	1.78^bc^	1.74^c^	1.82^abc^	0.03	0.018	0.033	0.006

^a–c^Means with different superscripts within the same row differ significantly (*p* < 0.05). BW, body weight; ADG, average daily gain; ADFI, average daily feed intake; F:G, feed to gain ratio.

### 3.2 Blood biochemical parameters

The effect of dietary supplementation with GP on blood biochemical indices is presented in Table 5. As evidenced by Table 5, on day 21, inclusion of 1,000, 1,500 and 2,000 mg/kg GP in the diet significantly elevated (*p* < 0.05) the activity of serum AKP. Supplementation at 1000 and 1,500 mg/kg GP significantly reduced (*p* < 0.05) the concentration of serum BUN. Furthermore, these effects exhibited both significant linear (*p* < 0.05) and quadratic (*p* < 0.05) dose–response relationships. On day 42, dietary supplementation with 1,000, 1,500 and 2,000 mg/kg GP markedly increased (*p* < 0.05) the concentrations of both GH and GLB. Moreover, the above effects exhibited a highly significant linear (*p* < 0.05) and quadratic (*p* < 0.05) increase with the augmentation of GP dosage, with the highest concentration showed in the 1,500 mg/kg group. Additionally, as the GP dosage increased, the concentrations of IGF-1 and BUN, respectively, showed a significant linear increase and decrease (*p* < 0.05).

**Table 5:** Effects of dietary GP on serum growth factors and biochemical parameters in broilers.

Items	Levels of dietary GP (mg/kg)					SEM	*p*-value		
	0	500	1,000	1,500	2,000		Treatment	Linear	Quadratic
21 d									
IGF-1 (μg/L)	17.57	17.98	18.60	20.43	20.62	1.42	0.442	0.055	0.163
GH (μg/L)	10.67	11.88	11.58	11.99	11.34	1.14	0.928	0.679	0.701
TP (μg/L)	50.63	51.30	52.65	54.55	54.78	2.36	0.649	0.111	0.290
GLB (g/L)	29.43	34.75	34.22	35.81	35.23	2.07	0.231	0.062	0.082
AST (U/L)	117.51	113.65	118.16	120.36	114.11	9.10	0.988	0.997	0.981
ALT (U/L)	31.14	33.11	32.97	32.57	33.38	1.82	0.915	0.475	0.722
AKP (U/L)	1634.98^b^	1708.32^ab^	1867.76^a^	1872.45^a^	1827.76^a^	59.86	0.042	0.010	0.009
BUN (mmol/L)	2.71^a^	2.27^ab^	1.61^b^	1.58^b^	1.95^ab^	0.28	0.049	0.026	0.010
42 d									
IGF-1 (μg/L)	17.07	19.24	18.04	20.65	20.55	1.09	0.126	0.022	0.075
GH (μg/L)	14.05^b^	17.03^ab^	18.31^a^	18.46^a^	18.17^a^	1.06	0.041	0.010	0.006
TP (μg/L)	44.95	46.76	47.10	49.38	45.68	2.71	0.812	0.625	0.582
GLB (g/L)	24.64^b^	26.67^ab^	28.51^a^	29.43^a^	29.18^a^	1.19	0.050	0.004	0.007
AST (U/L)	116.22	121 0.35	131.21	135.41	124.52	7.34	0.389	0.196	0.17
ALT (U/L)	31.43	33.90	34.26	35.46	33.18	1.63	0.518	0.328	0.221
AKP (U/L)	1556.79	1517.74	1564.71	1710.68	1684.81	100.99	0.593	0.154	0.349
BUN (mmol/L)	3.61	3.55	2.74	2.74	3.03	0.30	0.122	0.048	0.061

^a,b^Means with different superscripts within the same row differ significantly (*p* < 0.05). IGF1, insulin-like growth factor-1; GH, growth hormone; TP, total protein; GLB, globulin; AST, aspartate aminotransferase; ALT, alanine aminotransferase; AKP, alkaline phosphatase; BUN, blood urea nitrogen.

### 3.3 Body size and carcass traits

The effects of dietary supplementation with GP on body size and carcass traits of broilers are shown in Table 6, respectively. It was shown that on day 42, there were no statistically significant differences (*p* > 0.05) in body size and carcass traits among the different treatment groups.

**Table 6:** Effects of dietary GP on body size and carcass performance in broilers on day 42.

Items	Levels of dietary GP (mg/kg)					SEM	*p*-value		
	0	500	1,000	1,500	2,000		Treatment	Linear	Quadratic
Body size traits									
Body slant length(cm)	19.8	19.77	20.8	20.93	19.57	0.45	0.165	0.682	0.162
Tibial circumference(cm)	4.85	4.78	5.17	4.95	5.18	0.2	0.379	0.341	0.325
Tibial length(mm)	101.91	104.2	110.17	108.58	103.01	3.06	0.354	0.687	0.147
Hip bone width(mm)	78.93	79.6	80.61	78.57	77.15	1.35	0.502	0.302	0.2
Keel bone length(mm)	130.86	130.42	138.09	133.47	130.41	3.75	0.58	0.939	0.465
Chest depth(mm)	74.42	70.01	77.65	81.23	74.55	3.26	0.249	0.326	0.478
Chest width(mm)	80.49	85.19	85.88	91.85	80.53	2.13	0.073	0.198	0.395
Chest angle(degrees)	123.8	119.2	129.43	127.33	126.43	2.83	0.194	0.72	0.091
Carcass traits									
Fully eviscerated weight (g)	1454.7	1485.8	1,523	1527.7	1446.6	26.67	0.174	0.798	0.059
Semi-eviscerated weight (g)	1611.9	1622.8	1621.5	1,656	1614.3	30.49	0.845	0.678	0.752
Breast muscle ratio (%)	28.43	26.08	26.43	27.29	26.8	0.88	0.413	0.483	0.364
Thigh muscle ratio (%)	20.76	21.98	20.92	20.96	21.11	0.7	0.755	0.879	0.913
Abdominal fat ratio (%)	2.88	2.53	2.6	2.27	2.11	0.31	0.483	0.06	0.184
Lean meat ratio (%)	49.2	48.06	47.35	48.25	47.91	0.86	0.66	0.372	0.402

### 3.4 Meat quality parameters

#### 3.4.1 Sensory and physical quality indicators

The effects of dietary GP addition on the meat quality of broiler chicken breast and leg muscles are presented in Tables 7, 8, respectively. As shown in Table 7, with the increase in GP dosage, the pH value of the breast muscle at 24 h showed a significant linear increase (*p* < 0.05). The shear force exhibited a significant quadratic decrease (*p* < 0.05), while both the drip loss rate and cooking loss rate demonstrated significant linear (*p* < 0.05) and quadratic (*p* < 0.05) decreases. Moreover, compared to the control group and the 500 mg/kg GP group, the cooking loss rate was significantly reduced in the 1,000, 1,500 and 2,000 mg/kg GP groups (*p* < 0.05).

**Table 7:** Effects of dietary GP on pectoral meat color, pH, shear force, drip loss and cooking loss rates in broilers on day 42.

Items	Levels of dietary GP (mg/kg)					SEM	*p*-value		
	0	500	1,000	1,500	2,000		Treatment	Linear	Quadratic
Meat color^1^									
L	40.79	48.76	41.85	44.58	48.40	2.90	0.258	0.271	0.560
a	7.79	7.95	8.68	7.75	8.16	0.42	0.578	0.624	0.642
b	15.65	17.53	19.69	17.59	17.26	1.62	0.560	0.523	0.269
pH									
45 min	6.96	6.87	6.62	6.88	6.90	0.13	0.473	0.780	0.322
24 h	6.44	6.48	6.52	6.68	6.74	0.15	0.123	0.039	0.059
Shear force (N)	31.96	20.77	18.07	19.99	27.96	4.14	0.182	0.631	0.032
Drip loss rate (%)	8.89	7.11	6.58	6.06	6.25	0.01	0.081	0.010	0.012
Cooking loss rate (%)	34.33^a^	33.33^a^	27.33^b^	18.00^c^	20.33^c^	0.02	0.001	0.001	0.001

^a–c^Means with different superscripts within the same row differ significantly (*p* < 0.05). ^1^L, lightness; a, redness; b, yellowness.

**Table 8:** Effects of dietary GP on leg meat color, pH, shear force, drip loss and cooking loss rates in broilers on day 42.

Items	Levels of dietary GP (mg/kg)					SEM	*p*-value		
	0	500	1,000	1,500	2,000		Treatment	Linear	Quadratic
Meat color^1^									
L	51.76	50.01	53.66	62.32	66.53	6.56	0.398	0.050	0.119
a	14.85	13.58	13.30	15.78	11.65	1.48	0.465	0.304	0.564
b	18.87	18.88	17.25	19.67	13.60	1.95	0.209	0.107	0.183
pH									
45 min	7.39	7.19	7.08	7.19	7.08	0.11	0.336	0.081	0.132
24 h	7.34	7.40	7.67	7.63	7.45	0.12	0.329	0.122	0.513
Shear force (N)	12.23	10.63	11.91	10.46	15.12	1.76	0.431	0.278	0.218
Drip loss rate (%)	8.90^a^	7.34^b^	6.96^b^	6.76^b^	6.95^b^	0.01	0.036	0.011	0.005
Cooking loss rate(%)	35.35^a^	33.31^ab^	27.67^bc^	20.20^c^	24.31^c^	0.02	0.006	0.001	0.002

^a–c^Means with different superscripts within the same row differ significantly (*p* < 0.05). ^1^L, lightness; a, redness; b, yellowness.

Table 8 indicates that with the increase in GP addition, the brightness of leg muscles showed a significant linear increase (*p* < 0.05), while both the drip loss rate and cooking loss rate exhibited significant linear (*p* < 0.05) and quadratic (*p* < 0.05) decreases. Furthermore, compared to the control group, the drip loss rate was significantly reduced in the GP dosage groups (*p* < 0.05), although the reduction in cooking loss rate was not significant in the 500 mg/kg GP group (*p* > 0.05), while it was significantly reduced in the other dosage groups (*p* < 0.05).

#### 3.4.2 Muscle fiber characteristics

The effects of GP on muscle fiber characteristics are presented in Table 9. With increasing levels of dietary GP supplementation, a significant linear (*p* < 0.05) and quadratic (*p* < 0.05) decrease in the diameter of pectoral muscle fibers was shown. The density of the pectoral muscle fibers exhibited a significant linear (*p* < 0.05) and quadratic (*p* < 0.05) increase, with the groups receiving 1,500 and 2000 mg/kg GP showing a significant enhancement (*p* < 0.05) compared to the other groups. The diameter and density of leg muscle fibers did not show statistically significant differences (*p* > 0.05) among the various treatment groups.

**Table 9:** Effects of dietary GP on muscle fiber diameter and density in broilers on day 42.

Items	Levels of dietary GP (mg/kg)					SEM	*p*-value		
	0	500	1,000	1,500	2,000		Treatment	Linear	Quadratic
Pectoral muscle fiber									
Diameter (μm)	31.06	30.17	30.27	28.23	28.28	0.87	0.110	0.009	0.036
Density (/mm^2^)	906.3^b^	1010.6^b^	1004.1^b^	1297.5^a^	1291.0^a^	91.8	0.018	0.001	0.006
Leg Muscle Fiber									
Diameter (μm)	28.949	28.06	27.584	25.137	27.363	1.32	0.362	0.153	0.254
Density (/mm^2^)	1382.3	1480.1	1584.4	1682.2	1467.1	102.7	0.313	0.272	0.147

^a,b^Means with different superscripts within the same row differ significantly (*p* < 0.05).

#### 3.4.3 TBARS values, carbonyl and sulfhydryl content

**Figure 1:**
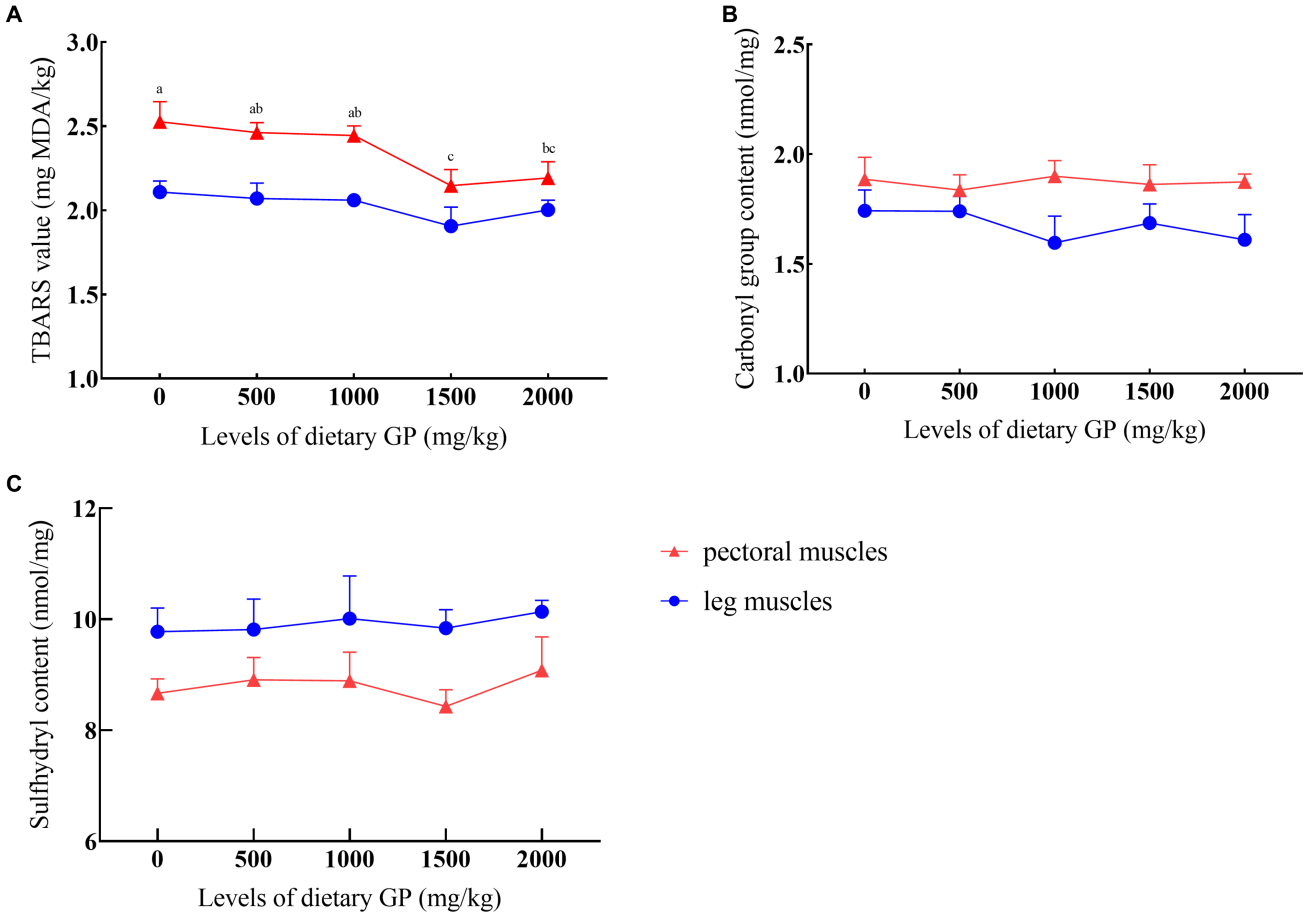
Effects of dietary GP on TBARS, carbonyl group, and sulfhydryl content over 90 days. **(A)** TBARS value, **(B)** Carboxyl group content, **(C)** Sulfhydryl content. Each value is shown as the mean and SEM (*n* = 5). ^a–c^Means with different superscripts within the same row differ significantly (*p* < 0.05).

The effects of GP levels on TBARS values, carbonyl, and sulfhydryl content are presented in Figure 1. It was shown that after 90 days of storage at −20°C, compared to the control group, the TBARS values significantly decreased (*p* < 0.05) in the groups supplemented with 1,500 and 2000 mg/kg GP, with the lowest TBARS values recorded in the 1,500 mg/kg GP group, significantly differing from both the 500 and 1,000 mg/kg GP groups (*p* < 0.05). GP did not exert a noticeable effect on the carbonyl and sulfhydryl content in both breast and leg muscles (*p* > 0.05).

#### 3.4.4 Fatty acid composition

The effects of GP on the fatty acid composition of pectoral and leg muscles are presented in Tables 10, 11, respectively. As shown in Table 10, GP significantly increased (*p* < 0.05) the content of C18:2n6 in the pectoral muscle, and significantly decreased (*p* < 0.05) the levels of C14:0, C18:0 and C22:6n3. These changes exhibited significant linear (*p* < 0.05) and quadratic effects (*p* < 0.05) with increasing doses of GP. The most notable effects were shown in the group supplemented with 1,500 mg/kg GP. Furthermore, with increasing GP doses, the pectoral muscle showed significant linear or quadratic increases (*p* < 0.05) in C16:0, C16:1, and C18:1n9c, while C12:0, C17:0, and C20:1 demonstrated significant linear or quadratic decreases (*p* < 0.05).

**Table 10:** Effects of dietary GP on the fatty acid composition of pectoral muscle in broilers (mg/100 g).

Items	Levels of dietary GP (mg/kg)					SEM	*p*-value		
	0	500	1,000	1,500	2,000		Treatment	Linear	Quadratic
C10:0	1.73	1.42	1.75	1.48	1.31	0.15	0.201	0.127	0.280
C12:0	1.14	0.81	0.85	0.77	0.81	0.10	0.078	0.036	0.028
C14:0	11.19^a^	9.88^ab^	9.42^b^	8.74^b^	9.67^ab^	0.49	0.031	0.019	0.006
C14:1	1.88	2.08	1.99	1.56	1.80	0.24	0.619	0.381	0.660
C15:0	2.61	2.44	2.38	2.32	2.35	0.13	0.556	0.121	0.210
C16:0	353.95	410.75	399.56	471.07	488.05	43.21	0.212	0.018	0.066
C16:1	46.53	47.14	57.85	58.60	55.56	5.53	0.374	<0.001	<0.001
C17:0	4.68	4.09	4.01	3.83	3.96	0.23	0.120	0.028	0.027
C18:0	172.75^a^	152.62^ab^	134.23^b^	124.78^b^	134.77^b^	9.47	0.015	0.003	0.002
C18:1n9t	2.67	3.23	2.76	3.25	2.57	0.62	0.897	0.928	0.797
C18:1n9c	596.19	627.89	661.67	719.89	712.81	34.25	0.081	0.004	0.017
C18:2n6	403.12^b^	481.16^a^	462.71^ab^	511.73^a^	496.95^a^	23.09	0.030	0.008	0.014
C18:3n3	29.72	34.29	31.42	33.04	33.65	1.97	0.490	0.292	0.523
C18:3n6	4.52	4.82	4.51	4.49	4.94	0.19	0.359	0.432	0.542
C20:0	2.43	2.43	2.45	2.30	2.37	0.10	0.834	0.432	0.739
C20:1	5.03	4.47	3.65	3.67	4.46	0.42	0.138	0.186	0.038
C20:2	3.50	3.39	3.33	3.32	3.14	0.19	0.769	0.484	0.505
C20:3n6	2.87	2.92	3.02	2.86	3.07	0.28	0.975	0.681	0.917
C20:4n6	25.64	29.91	26.40	31.68	27.72	1.59	0.078	0.302	0.308
C22:0	1.45	1.27	1.32	1.34	1.20	0.08	0.333	0.105	0.275
C22:6n3	8.57^a^	8.46^a^	7.80^ab^	6.93^b^	8.05^ab^	0.33	0.016	0.040	0.034
C24:0	1.23	1.25	1.29	1.20	1.28	0.07	0.894	0.752	0.952
C24:1	1.11	1.19	1.17	1.19	1.07	0.04	0.163	0.484	0.058
SFA (%)	32.69	32.01	30.22	30.85	32.10	1.75	0.854	0.664	0.560
PUFA (%)	67.31	67.99	69.78	69.15	67.90	1.75	0.854	0.664	0.560

^a–c^Means with different superscripts within the same row differ significantly (*p* < 0.05). The fatty acids are denoted as follows: C10:0, capric acid; C12:0, lauric acid; C14:0, myristic acid; C14:1, myristoleic acid; C15:0, pentadecanoic acid; C16:0, palmitic acid; C16:1, palmitoleic acid; C17:0, heptadecanoic acid; C18:0, stearic acid; C18:1n9t, elaidic acid; C18:1n9c, oleic acid; C18:2n6, linoleic acid; C18:3n3, alpha-linolenic acid; C18:3n6, gamma-linolenic acid; C20:0, arachidic acid; C20:1, eicosenoic acid; C20:2, eicosadienoic acid; C20:3n6, dihomo-gamma-linolenic acid; C20:4n6, arachidonic acid; C22:0, behenic acid; C22:6n3, docosahexaenoic acid; C24:0, lignoceric acid; C24:1, nervonic acid; SFA refers to saturated fatty acids and PUFA refers to polyunsaturated fatty acids. The same annotations apply to the following table.

**Table 11:** Effects of dietary GP on the fatty acid composition of leg muscle in broilers (mg/100 g).

Items	Levels of dietary GP (mg/kg)					SEM	*p*-value		
	0	500	1,000	1,500	2,000		Treatment	Linear	Quadratic
C10:0	4.83	4.68	5.19	4.30	4.11	0.41	0.365	0.162	0.236
C12:0	0.53	0.46	0.48	0.41	0.37	0.10	0.824	0.227	0.488
C14:0	15.13	13.72	14.21	12.84	12.97	0.74	0.212	0.032	0.096
C14:1	2.17	2.22	2.07	1.90	1.84	0.34	0.913	0.341	0.629
C15:0	2.35	2.55	2.54	2.60	2.57	0.14	0.747	0.257	0.404
C16:0	497.33	491.31	599.51	571.54	547.75	55.94	0.600	0.304	0.422
C16:1	49.11	48.66	48.04	54.06	52.75	5.40	0.907	0.439	0.723
C17:0	7.20	6.31	6.65	6.37	6.39	0.38	0.457	0.193	0.306
C18:0	300.53^a^	256.41^b^	261.14^b^	258.92^b^	245.88^b^	9.80	0.009	0.004	0.006
C18:1n9t	2.80	3.54	3.49	3.93	4.20	0.44	0.260	0.024	0.081
C18:1n9c	581.54	639.07	648.35	728.40	645.37	32.65	0.069	0.060	0.046
C18:2n6	765.29^b^	1008.97^a^	915.65^ab^	1012.69^a^	894.96^ab^	51.47	0.017	0.190	0.024
C18:3n3	40.61	42.84	40.85	39.55	43.33	2.80	0.859	0.804	0.906
C18:3n6	4.19	4.58	4.19	4.28	4.25	0.24	0.756	0.811	0.889
C20:0	2.50^a^	2.40^ab^	2.32^abc^	2.20^bc^	2.16^c^	0.07	0.019	0.000	0.002
C20:1	5.55	5.81	5.54	5.46	5.83	0.48	0.971	0.882	0.961
C20:2	3.24	3.45	3.08	2.93	2.94	0.20	0.357	0.831	0.978
C20:3n6	1.60	1.81	2.32	2.35	2.00	0.29	0.320	0.155	0.124
C20:4n6	28.06	27.85	27.75	27.25	29.51	2.01	0.946	0.705	0.766
C22:0	1.38	1.35	1.34	1.31	1.28	0.08	0.935	0.353	0.655
C22:6n3	18.15	17.11	16.63	15.57	16.16	0.81	0.254	0.035	0.076
C24:0	1.38	1.17	1.23	1.28	1.29	0.08	0.410	0.798	0.337
C24:1	3.78	3.68	3.83	3.88	3.39	0.45	0.942	0.665	0.787
SFA (%)	35.45	30.10	34.27	31.04	32.45	1.70	0.193	0.386	0.466
PUFA (%)	64.55	69.90	65.73	68.96	67.55	1.70	0.193	0.386	0.466

^a,b^Means with different superscripts within the same row differ significantly (*p* < 0.05).

As indicated in Table 11, GP significantly increased the content of C18:2n6 in the leg muscle and significantly decreased the levels of C18:0 and C20:0, with these changes also showing significant linear (*p* < 0.05) and quadratic effects (*p* < 0.05) as GP dosage increased. The most effective results were again seen in the 1,500 mg/kg GP group. Additionally, with increasing GP doses, the leg muscle exhibited significant linear or quadratic increases (*p* < 0.05) in C18:1n9t and C18:1n9c, whereas C14:0 and C22:6n3 showed significant linear decreases (*p* < 0.05). Overall, there were no significant differences (*p* > 0.05) in the percentages of Saturated Fatty Acids (SFA) and Polyunsaturated Fatty Acids (PUFA) between the groups in both pectoral and leg muscles.

### 3.5 Gene expression

**Figure 2:**
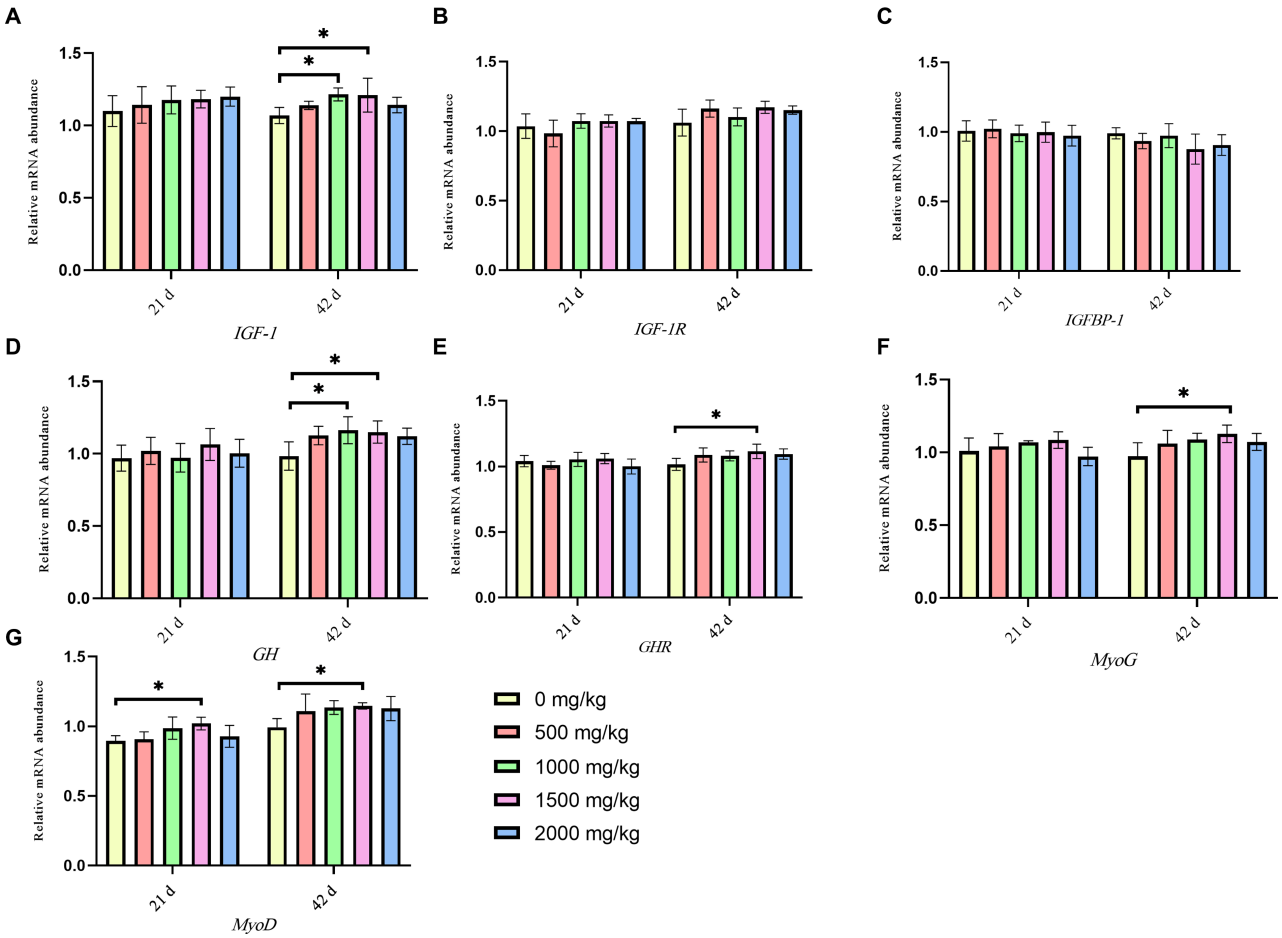
Effects of dietary GP on the relative gene expression level of growth and meat quality-related gene of leg muscle in broilers on day 42. **(A)** *IGF-1* expression, **(B)** *IGF-1R* expression, **(C)** *IGFBP-1* expression, **(D)** *GH* expression, **(E)** *GHR* expression, **(F)** *MyoG* expression, **(G)** *MyoD* expression. Each value is shown as the mean and SEM (n = 5). Data columns with * mean a significant difference between groups (*p* < 0.05). *IGF-1*, insulin-like growth factor-1; *IGF-1R,* insulin-like growth factor 1 receptor; *IGFBP-1*, insulin like growth factor binding protein 1; *GH*, growth hormone; *GHR*, growth hormone receptor; *MyoG*, myogenin; *MyoD*, myogenic differentiation.

Figure 2 present the effects of GP on the expression levels of relevant genes. On day 21, compared to the control group, the 1,500 mg/kg GP group exhibited a significant increase in the relative expression level of *MyoD* mRNA (*p* < 0.05), while there were no significant changes in the mRNA relative expression levels of other genes (*p* > 0.05). On day 42, significant increases were shown in the relative expression levels of *GH* and *IGF-1* mRNA in the 1,000 mg/kg GP group (*p* < 0.05) and in the levels of *GH*, *GHR*, *MYoG*, *MyoD* and *IGF-1* mRNA in the 1,500 mg/kg GP group (*p* < 0.05). There were no statistically significant differences in the relative expression levels of *IGF-1R* and *IGFBP-1* mRNA among the groups (*p* > 0.05).

## 4 Discussion

In animal husbandry, enhanced growth performance is directly linked to increased production (28). Various plant-derived polysaccharides have been demonstrated to positively influence animal growth. For instance, Ao and Kim showed that including *Achyranthes bidentata* polysaccharide in the diet of Pekin ducks notably enhanced feed efficiency and growth performance between days 22 to 42 and from day 1 to 42 (29). Wang et al. (30) found that supplementing the diets of weaned piglets with 800 mg/kg of ginseng polysaccharide significantly improved their growth performance and feed utilization rate. However, contrasting findings were reported by Chen et al. (31), who found that the dietary addition of Achyranthan and Astragalan had no effect on the production performance in broilers. These findings suggest that the effects of plant polysaccharides on animal growth may vary depending on the type of polysaccharide and the animal species.

In this study, during the intervals of 22 to 42 days and from 1 to 42 days, the dietary inclusion of GP at concentrations of 1,000 and 1,500 mg/kg significantly reduced the F:G ratio. This finding aligns with the results obtained by Zhang et al. (16). However, diverging from previous research, our results indicated that there was no significant difference in the growth performance of broilers in the group receiving 2,000 mg/kg GP compared to the control group. This suggests a threshold effect where increased concentrations beyond 1,500 mg/kg do not confer additional benefits in growth performance. Previous studies suggest that the enhancement of growth performance in broilers by herbal polysaccharides might be achieved through increasing the digestibility of dry matter and nitrogen (32), or by enhancing the activity of digestive enzymes (33). Alteration of the intestinal microbial structure (34) may also play a role. However, the results of this study indicate that this effect might be related to increased concentrations of GH and IGF-1, as well as the enhanced expression of genes associated with growth.

The neuroendocrine growth axis “hypothalamus-pituitary-growth hormone-target organs” regulated the growth and development of poultry (35). Extant research indicated that the somatotropic axis hormones, including growth hormone-releasing hormone (GHRH), IGFs, GH and somatostatin, predominantly coordinated animal growth (36). The *GH* gene could regulate the growth and development of poultry through the growth axis and could also interact with downstream molecules to form the GH-GHR-IGF-I pathway, thereby affecting the growth and development of various tissues and organs in animals (37). GH was also produced by other tissues and this GH acted locally on IGF-1 in tissues or organs through autocrine or paracrine mechanisms (38). GHR is a membrane protein that binds specifically and efficiently with GH. The interaction between GH and GHR was essential for GH to exert its effects, representing a key mechanism in the functioning of GH (39). The *IGF-1* gene was involved in activating RNA polymerase, thereby regulating the synthesis of RNA and DNA, promoting cell proliferation and differentiation and stimulating the growth of muscle and bone cells. The *IGF-1* gene played a critical role in the growth and development of the body and was one of the essential growth regulatory factors (40). This study demonstrated that with an increase in GP dosage, the concentrations of GH and IGF-1 in the serum at 42 days exhibited a significant linear rise. Compared to the control group, there was a marked increase in the expression levels of *GH* and *IGF-1* genes in the leg muscle in groups supplemented with 1,000 and 1,500 mg/kg GP, as well as an elevated expression of *GHR* mRNA in the leg muscle of the 1,500 mg/kg GP group. As indicated by Young et al., skeletal muscle is a primary target for GH (and IGF-1), with growth-promoting effects (41). Therefore, in this trial, the enhanced expression of *GH* and *IGF-1* genes, leading to increased secretion of GH and IGF-1, was associated with improved growth performance in broilers. Additionally, as noted by Segard et al. (42), autocrine/paracrine GH has been linked to muscle cell proliferation and myotube differentiation. This concept elucidates the observed increase in pectoral muscle fiber density in this experiment, which can be attributed to the elevated expression and concentration of GH mRNA, fostering the proliferation of muscle cells.

Research indicated that increased TP and albumin levels, along with enhanced activities of AST, ALT and AKP, were markers of high protein metabolism in the body (43, 44). Conversely, BUN levels had been found to inversely correlate with protein synthesis (45). In this trial, the TP levels and ALT and AST activities in the group treated with GP had increased to varying degrees, but without significant differences. However, the activity of AKP had significantly increased, and BUN levels had significantly decreased in the groups receiving more than 1,000 mg/kg of GP at 21 d, with the most pronounced effect showed in the 1,500 mg/kg GP group. These effects were not significantly different at 42 d. The results indicated that the effects of GP on protein metabolism and synthesis in broilers predominantly occurred in the early stages of growth. Previous research had demonstrated that the efficiency of protein synthesis in broilers was higher during the 0–3-week period compared to the 4–6-week period, overall showing a pattern of initial linear increase followed by a plateau (46).

Additionally, as a constituent of total serum protein, the level of GLB could reflect the immune status of the organism (47). At 42 d of the trial, when the GP supplementation exceeded 1,000 mg/kg, there was a significant increase in GLB concentration, suggesting that GP might affect the immune function of broilers in the later stages of growth. Zhou et al. had reported that GP significantly enhanced the serum antibody titer in Lohmann Brown chickens, promoted the development of immune organs and proliferation of immune cells and stimulated lymphocytes and dendritic cells to secrete relevant cytokines (19). Wu et al. (48) demonstrated that the synergistic effect of GP with the NDV vaccine in chickens led to the production of an increased number of Newcastle disease antibodies, which held significant importance in the prevention of Newcastle disease. Additionally, previous research has demonstrated that GP exerted beneficial effects on the antibody titer, phagocytosis index, and cecal microflora in broilers (49).

In the present experiment, although GP did not affect the body size and carcass traits of broilers, it positively influenced their meat quality. Meat color, pH value, shear force, drip loss and cooking loss were identified as critical parameters for assessing meat quality (50). Our experiment showed that with an increase in the dosage of GP, the pH of breast muscle at 24 h showed a significant linear increase, while shear force, drip loss and cooking loss each exhibited significant linear or quadratic decreases. Similarly, the pH of leg muscle at 24 h tended to increase, but this data did not show significant differences. Moreover, leg muscle drip loss and cooking loss also demonstrated the aforementioned significant differences. To date, no reports have been found regarding the impact of GP on chicken meat quality. However, related research on plant polysaccharides indicated that *Yingshan yunwu* green tea polysaccharide could increase the postmortem pH value of chicken breast meat and decrease its acidification (51). Additionally, Chang et al. reported that the addition of Chinese yam polysaccharide to the diet reduced the shear force of broiler meat post-slaughter (52). This was similar to the conclusions drawn from our experiment. Wang et al. (53) showed that the ability of muscle proteins to attract and retain water within the cells was crucial for meat quality. Generally, low pH reduced the ability of muscle proteins to bind water and decreased the negative electrostatic repulsion between filaments. This reduction in space between filaments led to myofibril shrinkage (54). The experiment showed that GP significantly decreased the diameter and increased the density of pectoral muscle fibers. This effect was attributed to an increase in the pH value of the pectoral muscle, aligning with previous findings. Related research indicated that lower shear force and moisture loss rate were associated with better meat quality, characterized by finer muscle fibers and higher muscle water content (55–57). Furthermore, muscles with smaller fiber diameters and higher densities were known to have superior sensory tenderness (58).

*MyoD* and *MyoG* genes, both members of the *MRFs* family, were found to be involved in regulating the number and size of muscle fibers, as well as muscle cell proliferation and differentiation (59). *MyoD* was shown to activate the transcription and expression of various myogenic factors, thereby contributing to the development of skeletal muscle (60). Similarly, *MyoG* was instrumental in enhancing cellular differentiation and facilitating the formation of skeletal muscle fibers (61). Research has shown that smaller diameters of muscle fibers significantly enhanced meat quality attributes such as tenderness, water-holding capacity, flavor and juiciness (62, 63). Accordingly, a greater density of muscle fibers led to a finer meat texture and improved palatability. In the current experiment, the group treated with 1,500 mg/kg GP showed increased expressions of *MyoD* mRNA at 21 d and both *MyoG* and *MyoD* mRNA at 42 d. Correspondingly, the meat quality in this group was shown to be superior. These findings suggest that changes in shear force, drip loss, cooking loss and the physical properties of muscle fibers, such as diameter and density, were regulated by the *MyoD* and *MyoG* genes. However, research by Aguiar et al. indicated that while chronic low-frequency electrical stimulation influenced muscle fiber diameter and density, it did not significantly alter the mRNA expression levels of *MyoD* and *MyoG* (64). This discrepancy underscores the need for further research to fully understand the effects of GP on broiler meat quality and the underlying regulatory mechanisms.

TBARS is a major indicator of lipid oxidation in meat and meat products (65). Protein carbonyls and free sulfhydryl groups are parameters for measuring protein oxidation, often accompanied by an increase in carbonyl values and a decrease in free sulfhydryl groups (66). The results of this experiment showed that, compared to the control group, the TBARS values in the pectoral muscle significantly decreased in the groups supplemented with 1,500 and 2000 mg/kg GP, indicating that GP inhibited lipid oxidation. It is currently believed that this result is due to the suppression of hydroperoxide production (67). Previous studies have demonstrated that GP has a notable ability to scavenge free radicals, including DPPH, hydrogen peroxide, ABTS, and superoxide anion radicals (19), which well explains our findings. Notably, the maximum TBARS value in fresh meat is 1.0 mg MDA/kg (68), yet the shown TBARS values in our study were all above 2.0 mg MDA/kg. This could be due to certain limitations, as our experiment only measured chicken meat samples stored at −20°C for 90 days.

Fatty acids in muscle tissue serve as critical indicators for assessing the nutritional value and contribute to the understanding of the flavor profile and nutritional importance (69). The results of this study revealed that the inclusion of GP in the feed of broilers significantly altered the composition of fatty acids in both pectoral and leg muscles. Specifically, there was an increase in the levels of linoleic acid (C18:2n6) and oleic acid (C18:1n9c), coupled with a decrease in the concentrations of stearic acid (C18:0), myristic acid (C14:0), and docosahexaenoic acid (C22:6n3). Studies have identified myristic acid as having hypercholesterolemic effects, potentially serving as a precursor to coronary diseases (70), suggesting the potential of GP in reducing hyperlipidemia. Docosahexaenoic acid, when present at elevated levels in meat, can catalyze lipid peroxidation, compromising meat quality and preservation. However, its role as a vital functional fatty acid for brain, retinal, skin, and renal health, and its association with cardiovascular disease prevention and cognitive performance enhancement in young adults, underscores its nutritional significance (71). In this experiment, the supplementation with GP led to a significant increase in the levels of docosahexaenoic acid in both pectoral and leg muscles, possibly due to GP’s antioxidant capacity, which may mitigate the generation of lipid peroxides by scavenging free radicals. The observed decrease in stearic acid alongside the increase in oleic acid may result from the rapid bioconversion of stearic acid into oleic acid (72). While oleic acid is recognized for its role in preventing cardiovascular and cerebrovascular diseases through the modulation of lipoprotein levels (73), stearic acid is suggested to enhance hedonic feeding behaviors and mesolimbic dopamine signaling in animal models (74). Overall, the research provided evidence that GP, without significantly changing the ratio of saturated to PUFA in chicken meat, can modulate the fatty acid metabolism within chicken muscle tissues. The findings lay a foundation for further investigations into the mechanistic actions of GP and its prospective applications in augmenting the quality and health benefits of broiler meat.

## 5 Conclusion

In this study, dietary supplementation with GP effectively reduced feed conversion rates, enhanced growth hormone concentrations, improved biochemical blood parameters, and upregulated growth-associated genes in broilers. GP also significantly lowered TBARS values in stored chicken meat, suggesting an extended shelf life, and altered fatty acid composition and meat quality traits, with changes in meat quality likely associated with the regulation of *MyoD* and *MyoG* genes. Optimal results were achieved with 1,500 mg/kg GP, highlighting its potential as a beneficial feed additive for enhancing broiler growth and meat quality.

## Data availability statement

The datasets presented in this study can be found in online repositories. The names of the repository/repositories and accession number(s) can be found in the article/supplementary material.

## Ethics statement

The animal study was approved by Animal Care and Use Committee of the College of Animal Science and Technology of Inner Mongolia Minzu University. The study was conducted in accordance with the local legislation and institutional requirements.

## Author contributions

TL: Funding acquisition, Formal analysis, Software, Visualization, Writing – original draft. WQ: Project administration, Supervision, Writing – original draft. BW: Investigation, Software, Writing – original draft. XJ: Conceptualization, Writing – original draft. RZ: Methodology, Writing – original draft. JZ: Software, Writing – original draft. LD: Funding acquisition, Project administration, Supervision, Writing – review & editing.
